# Patterns of IgE responses to multiple allergen components and clinical symptoms at age 11 years

**DOI:** 10.1016/j.jaci.2015.03.027

**Published:** 2015-11

**Authors:** Angela Simpson, Nevena Lazic, Danielle C.M. Belgrave, Phil Johnson, Christopher Bishop, Clare Mills, Adnan Custovic

**Affiliations:** a Centre for Respiratory Medicine and Allergy, Institute of Inflammation and Repair, Manchester Academic Health Science Centre, University of Manchester & University Hospital of South Manchester, Manchester, United Kingdom; b Microsoft Research Cambridge, Cambridge, United Kingdom; c Centre for Health Informatics, Institute of Population Health, University of Manchester, Manchester, United Kingdom; d Centre for Respiratory Medicine and Allergy, Institute of Inflammation and Repair, Manchester Institute of Biotechnology, University of Manchester, Manchester, United Kingdom

**Keywords:** IgE, childhood, component-resolved diagnostics, latent variable modeling, allergens, asthma, wheeze, eczema, hay fever, CG, Component group, CRD, Component-resolved diagnostics, eNO, Exhaled nitric oxide, ISAC, Immuno Solid-phase Allergen Chip, ISU, ISAC standardized units, OR, Odds ratio, sIgE, Serum IgE

## Abstract

**Background:**

The relationship between sensitization to allergens and disease is complex.

**Objective:**

We sought to identify patterns of response to a broad range of allergen components and investigate associations with asthma, eczema, and hay fever.

**Methods:**

Serum specific IgE levels to 112 allergen components were measured by using a multiplex array (Immuno Solid-phase Allergen Chip) in a population-based birth cohort. Latent variable modeling was used to identify underlying patterns of component-specific IgE responses; these patterns were then related to asthma, eczema, and hay fever.

**Results:**

Two hundred twenty-one of 461 children had IgE to 1 or more components. Seventy-one of the 112 components were recognized by 3 or more children. By using latent variable modeling, 61 allergen components clustered into 3 component groups (CG1, CG2, and CG3); protein families within each CG were exclusive to that group. CG1 comprised 27 components from 8 plant protein families. CG2 comprised 7 components of mite allergens from 3 protein families. CG3 included 27 components of plant, animal, and fungal origin from 12 protein families. Each CG included components from different biological sources with structural homology and also nonhomologous proteins arising from the same biological source. Sensitization to CG3 was most strongly associated with asthma (odds ratio [OR], 8.20; 95% CI, 3.49-19.24; *P* < .001) and lower FEV_1_ (*P* < .001). Sensitization to CG1 was associated with hay fever (OR, 12.79; 95% CI, 6.84-23.90; *P* < .001). Sensitization to CG2 was associated with both asthma (OR, 3.60; 95% CI, 2.05-6.29) and hay fever (OR, 2.52; 95% CI, 1.38-4.61).

**Conclusions:**

Latent variable modeling with a large number of allergen components identified 3 patterns of IgE responses, each including different protein families. In 11-year-old children the pattern of response to components of multiple allergens appeared to be associated with current asthma and hay fever but not eczema.

Although the presence of specific IgE (sIgE) to allergens is a major risk factor for asthma and hay fever, the relationship is inconsistent, and IgE-mediated sensitization is neither necessary nor sufficient for the expression of disease.^1^ In clinical practice and research studies patients are usually assigned as being atopic or not based on the results of skin prick or sIgE tests to extracts made from whole allergen sources.^2^, ^3^ One potential limitation of using whole-allergen extracts is that the sources used for their preparation contain multiple different allergenic proteins and that a positive result might reflect cross-reactivity consequent to homology between similar proteins in different allergen sources.^4^ The advent of molecular allergology has enabled investigators to identify individual proteins within whole allergen sources and to detect sIgE to individual allergen components.^5^ In patients with food allergy, measuring sensitization to components is more informative than measuring levels of IgE to whole extracts.^4^, ^6^, ^7^, ^8^ For some allergen sources, there is a dominant component to which most sensitized subjects will react (eg, Fel d 1 is positive in almost all subjects with IgE to cat), but for others, no such dominant allergen exists (eg, sensitization to Can f 1, 2, 3, and 5 identifies less than half of those with IgE to dog).^9^

The commercialization of molecular or component-resolved diagnostics (CRD) has facilitated the development of products in which sIgE to more than 100 allergen components can be measured simultaneously by using small volumes of serum.^10^, ^11^ One such technology is the multiplex Immuno Solid-phase Allergen Chip (ImmunoCAP Immuno Solid-phase Allergen Chip [ISAC]).^12^ We have recently reported that ISAC data might facilitate better assessment of allergic airway diseases.^13^

The role of such “high-resolution” tools in clinical practice and how best to interpret the complex data they generate is a matter of some debate.^14^, ^15^ Conventional analyses can overaggregate the underlying complexity^16^ and do not capture the heterogeneity in patterns of responses to multiple components, and therefore more sophisticated approaches are needed. A particularly appealing framework is that of latent variable models, in which a latent underlying mechanism explains the presence of multiple correlated items.^17^

We hypothesize that distinct patterns of component-specific IgE are associated with different clinical presentations. We propose that latent variable models can be used to identify such patterns and might facilitate better understanding of how data on sIgE to multiple allergen components can be interpreted within individual patients. To address our hypotheses, we measured levels of sIgE to 112 allergen components using a commercially available multiplex array in a population-based birth cohort and used a latent variable model to identify underlying patterns of component-specific IgE responses; these patterns were then related to asthma, eczema, and hay fever.

## Methods

### Study population

The Manchester Asthma and Allergy Study is a population-based birth cohort.^18^, ^19^, ^20^, ^21^, ^22^ Subjects were recruited prenatally and followed prospectively. The study was reviewed by the local institutional review board, and parents provided written informed consent. We used data collected at follow-up at age 11 years for this study. Validated questionnaires were interviewer administered to collect information on parentally reported symptoms, physician-diagnosed diseases, and treatments received.^23^ We performed spirometry and measured exhaled nitric oxide (eNO) levels and assessed airway hyperreactivity in a 5-step methacholine challenge test.^24^

### Definition of clinical outcomes

*Current wheeze* was defined as a positive answer to the following question: “Has your child had wheezing or whistling in the chest in the last 12 months?”^25^

*Current asthma* was defined as a positive answer to 2 of 3 of the following questions: “Has the doctor ever told you that your child had asthma?”; “Has your child had wheezing or whistling in the chest in the last 12 months?”; and “Has your child had asthma treatment in the last 12 months?”^26^

*Current hay fever* was defined as a positive answer to the following question: “Does your child have hay fever now?”^27^

*Current eczema* was defined as a positive answer to the following question: “Has your child had an itchy rash that comes and goes in the last 12 months?”^25^

*Lung function* was recorded as FEV_1_ and forced vital capacity values.^28^ Data were expressed as percent predicted FEV_1_^29^ and the FEV_1_/forced vital capacity ratio.

*Airway hyperreactivity* was defined as a greater than 20% decrease in FEV_1_ by the final stage of the challenge (16 mg/mL). We also calculated he dose-response slope to analyze the data as a continuous variable.^19^

*eNO* was recorded as a continuous variable in parts per billion.

### CRD

We measured levels of sIgE to 112 allergenic molecules (components) from 51 sources using ImmunoCAP ISAC (Thermo Fisher Scientific, Uppsala, Sweden). Levels of component-specific IgE antibodies were reported in ISAC standardized units (ISU). We transformed (discretized) sIgE data using a binary threshold of 0.3 ISU into 4 categories, according to the manufacturer's guidelines: no (<0.3 ISU), low (0.3-1 ISU), medium (1-15 ISU), and high (>15 ISU) sensitization.

### Statistical grouping of allergen components

Our statistical model assumed that there exist clusters of allergen components to which subjects have a similar IgE responses (ie, either being sensitized or not to most of the components within the same cluster). We refer to these clusters as component groups (CGs). We restricted our analysis to 71 components for which there were at least 3 children with positive test results. Components to which fewer than 3 children were sensitized are listed in Table E1 in this article's Online Repository at www.jacionline.org. We modeled different patterns of IgE response in 221 children with any positive test results.

We inferred allergen clusters and child sensitization to each cluster using Expectation Propagation,^30^ an algorithm for approximate Bayesian inference (see a detailed description in Fig E1 in this article's Online Repository at www.jacionline.org). We relied on the Expectation Propagation implementation (available in Infer.NET http://research.microsoft.com/infernet), a Microsoft-owned library for large-scale Bayesian inference freely available for research purposes. Inference was performed based solely on IgE responses without using any information about protein structure or function or clinical phenotypes. We assigned a component to a CG if the posterior cluster membership probability was greater than 0.7 and characterized each child as sensitized to a CG if the posterior sensitization probability was greater than 0.5. Robustness and reproducibility of the results were assessed by repeating the analysis on 20 random subsets of 200 subjects.

### Sequence, structure, and function of allergen components within CGs

We then investigated the sequence, structure, and functional properties of components clustered within the inferred CGs. We compiled an allergen sequence database (available on request) using allergens listed in the ALLFAM database (http://www.meduniwien.ac.at/allergens/allfam; release 2011-09-12)^31^ downloaded from the UniProt database (http://www.uniprot.org). Allergen sequences were grouped by Pfam clan/family designation and aligned with ClustalW2^32^ by using default settings. Dendrograms (average distance, BLOSUM62) were drawn by using Jalview, version 2.7. Dendrograms were colored and annotated by using Figtree, version 1.3.1 (http://tree.bio.ed.ac.uk/software/figtree). Within each protein family within a CG where 3 or more family members were present, we used a Venn diagram to map the distribution of sensitization.

### Associations between CGs and clinical outcomes

Each child was assigned a probability of being sensitized to each CG on a scale of 0 to 1. We then modeled the quantitative CG scores for each participant as a latent variable predictor of clinical outcome using multivariate logistic regression tests. In addition, we assigned each child as sensitized or not to each of the CGs by using a posterior cutoff threshold of 0.5 or greater, noting that each child could be sensitized to any combination of CGs, to quantify the prevalence of sensitization to each CG and provide descriptive statistics of the characteristics of children sensitized to each CG. Analyses were performed with Stata 12.1 and SPSS 20 software (IBM, Armonk, NY).

## Results

### Participant flow and demographic data

Among 1184 children born into the cohort, 822 attended follow-up at age 11 years; of these, CRD data were obtained for 461 (56.1%). There were no significant differences in demographic characteristics between children with and without CRD data (see Table E2 in this article's Online Repository at www.jacionline.org). Of 461 children included in this study, 221 (47.9%) had a positive sIgE result to at least one of the 112 allergen components, and 58 (12.5%) had asthma.

### Characteristics of CGs

**Fig 1:**
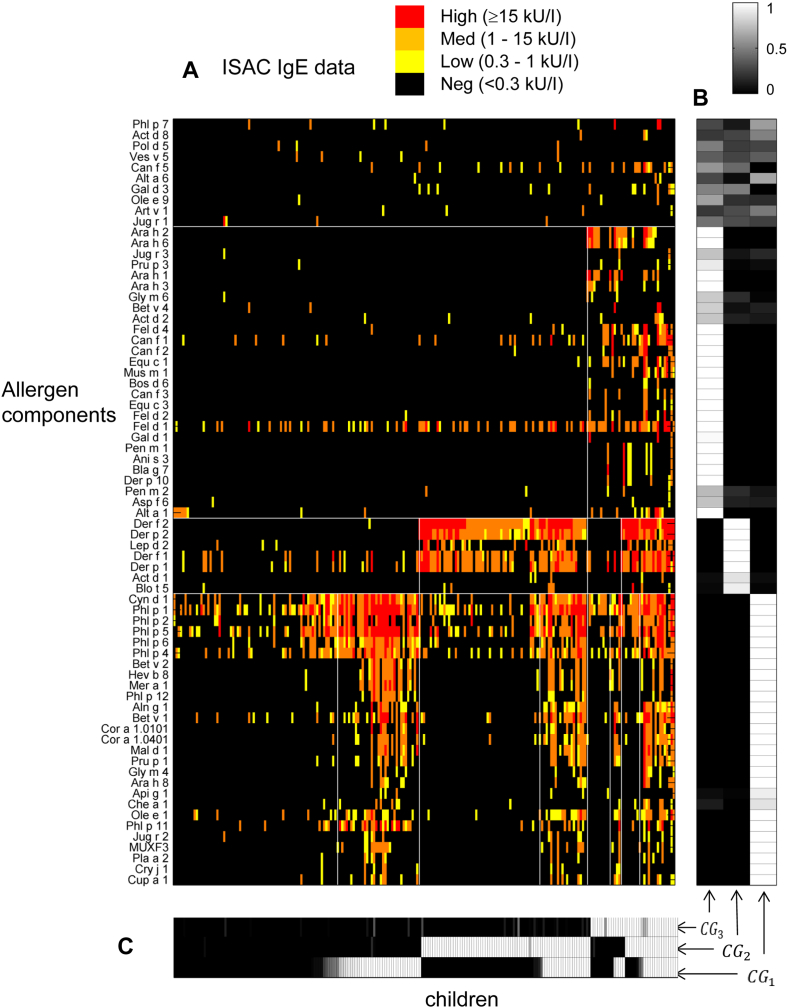
**A,** Specific IgE for each child to each allergen (quaternary scale, color coded: *black*, negative; *yellow*, low; *orange*, medium; and *red*, high). Allergen components are sorted according to CG membership, and children are sorted according to CG sensitization. **B,** Probability of each allergen component belonging to each CG *(grayscale)* based on the posterior distribution, indicating that for the 61 allergen components assigned to a CG, the probability was generally high (*white*, usually >0.9) **C,** Probability of each child being sensitized to each of the CGs *(grayscale)* based on the posterior sensitization probabilities, showing that children were frequently sensitized to more than 1 CG and that all combinations of sensitization were seen.

Of the 71 allergen components included in the model, 61 clustered into 3 groups (CG1, CG2, and CG3; Fig 1, *A*, and see Table E3 in this article's Online Repository at www.jacionline.org). The remaining 10 components did not strongly belong to any cluster, and therefore we did not assign these to any CG (non-CG components, see Table E3). The posterior distribution used to assign components to groups is shown in Fig 1, *B*. Most of the components were assigned to a single group with very high probability (>0.9). Group membership was very stable, as demonstrated by the component similarity matrix in Fig E2 in this article's Online Repository at www.jacionline.org. Repeating the analysis using quaternary data produced similar results, with CG assignment differing in only 3 components (see Table E4 in this article's Online Repository at www.jacionline.org). However, group membership was less stable (see Fig E3 in this article's Online Repository at www.jacionline.org). Therefore we opted for the binary data representation because it was sufficient to find patterns in the data, while requiring fewer model parameters.

The allergen components within each CG were then sorted by protein family (Pfam), as presented in Table I^33^ (see detailed accession numbers Table E5 in this article's Online Repository at www.jacionline.org). CG1 comprised 27 components exclusively of plant origin belonging to 8 different protein families. CG2 comprised 7 components almost exclusively made up of mite allergens classified into 3 protein families. CG3 was the most complex and included 27 components of plant, animal, and fungal origin drawn from 12 protein families. Of note, the protein families within each CG were exclusive to that group.

Ten non-CG allergens (to which there were generally fewer positive test results) came from 9 protein families; 2 were venom allergens, and 5 were from protein families not otherwise represented on the microarray. The 3 remaining allergens in the non-CG group are from protein families that already fall within CGs but to which very few children reacted.

### Sequence and structure of allergen components within CGs

Unrooted dendrograms of allergens belonging to different protein families within CGs and accompanying Venn diagrams mapping the distribution of sensitization to each component within each protein family (when ≥3 family members were present) are presented in Figs E4 to E8 in this article's Online Repository at www.jacionline.org. For some protein families, almost all subjects were sensitized to a single dominant allergen. Within the profilin family, the dominant allergens were Mer a 1 and Hev b 8, which bound IgE from all profilin-reactive subjects (see Fig E4). For the expansin family, Phl p 1 was the dominant allergen (see Fig E5). For the Bet v 1–like family, all but 1 child was sensitized to Bet v 1 (see Fig E6). For pectate lyase, only 3 of the 8 family members are represented on ISAC, and most subjects were sensitized to Cup a 1 (see Fig E7).

Similar relationships were observed for the CG2 components, with the cysteine proteases being dominated by Der p 1, whereas Der f 2 was the dominant allergen for the MD-2–related lipid recognition domain (ML domain) proteins (see Fig E9 in this article's Online Repository at www.jacionline.org).

For CG3 (see Fig E10 in this article's Online Repository at www.jacionline.org), the cupin allergen family was dominated by Ara h 1. For the prolamin family, Ara h 2 appeared to predominate, and for the serum albumins, it was Fel d 2. Fel d 1 was a frequent sensitizer in CG3, but it is the only member of the uteroglobulin-like family identified as an allergen. A dominant allergen was less likely to be identifiable where few children were sensitized to that protein family. Although few children were sensitized to tropomyosins, most children were sensitized to most allergens, without a clear dominant allergen. For the lipocalins, the overlapping sensitization patterns were not clear, although Can f 1 was the most common sensitizing allergen.

### Relationship between CG sensitization and clinical outcomes

**Fig 2:**
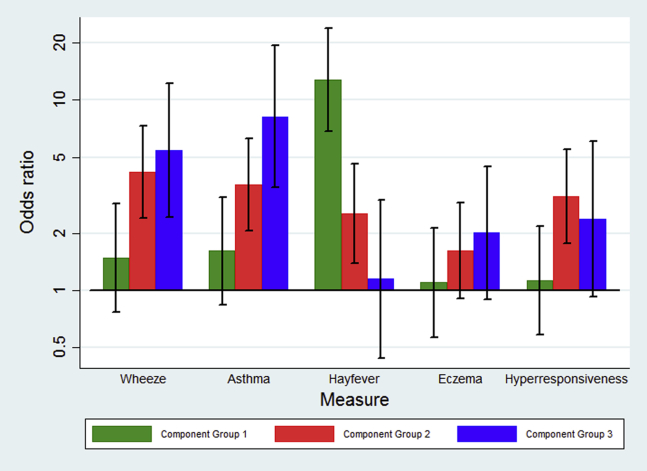
Association between sensitization to CGs and clinical outcomes. *Error bars* represent 95% CIs of the OR shown on a log scale.

We investigated the association between sensitization to each CG and clinical outcomes using the quantitative CG scores in a multivariate analysis (Fig 2 and Table II). Children sensitized to CG3 were more likely to have asthma (odds ratio [OR], 8.20; 95% CI, 3.49-19.24; *P* < .001), as were children sensitized to CG2 (OR, 3.60; 95% CI, 2.05-6.29; *P* < .001) but not CG1. A similar pattern was seen for wheeze. For hay fever, sensitization to CG1 was the strongest predictor (OR, 12.79; 95% CI, 6.84-23.90; *P* < .001), with a smaller effect seen for sensitization to CG2. For eczema, there was no significant association between sensitization to any of the CGs.

**Fig 3:**
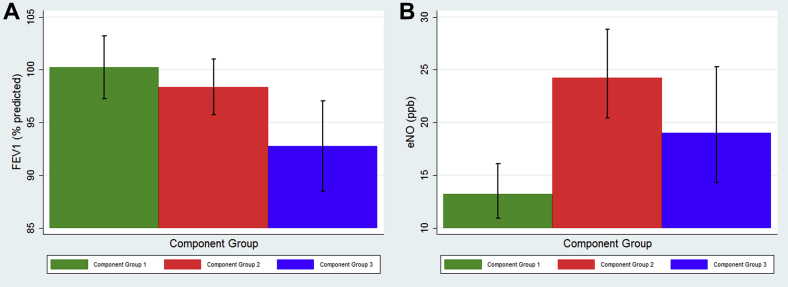
Estimated marginal means for FEV_1_ (percent predicted; **A**) and eNO **(B)** in those sensitized to CGs.

For lung function, only children sensitized to CG3 had a significantly poorer FEV_1_ percent predicted, with values in this group on average 6.8% lower than in children not sensitized to CG3 (95% CI, 2.69-11.06; *P* = .001; Fig 3, *A*). Airway reactivity was significantly greater in those sensitized to CG2, whether expressed as a dose/response ratio or a positive or negative methacholine challenge result (Fig 2). eNO levels were significantly higher among children sensitized to each of the CGs (*P* < .001; Fig 3, *B*).

We then assigned each child as sensitized or not to each of the CGs using a posterior cutoff threshold of 0.5 or greater. Of the 149 children who were sensitized to 1 or more CGs, 99 were sensitized to only 1, 34 were sensitized to 2, and 16 were sensitized to all 3 CGs (see Fig E11 and Tables E6 and E7 in this article's Online Repository at www.jacionline.org). For the nonsensitized group, two thirds of children were disease free, and eczema was numerically the most common disease (21.4%). For those sensitized to CG3, 28.6% had asthma, eczema, and hay fever as comorbidities, and only 2 (5.7%) children were disease free.

## Discussion

We demonstrated that different patterns of IgE responses to multiple allergen components can be uncovered using latent variable modeling and that each pattern bears different associations with clinical outcomes. Within those children with IgE responses, the allergen components to which they had IgE fell into 3 major clusters. The clustering was robust, reproducible, and biologically plausible. The review of the protein families to which the allergen components within each CG belonged indicated striking patterns, with each protein family being exclusive to only 1 CG. (Of note, the algorithms by which CGs were identified did not impose any prior clinical knowledge/information on protein sequence or clinical phenotypes of children.) Importantly, although children could be sensitized to more than 1 CG (and frequently were), sensitization to each distinct cluster (or CG) was associated with different patterns of disease. These results are consistent with our recent findings, which suggested the existence of multiple atopy phenotypes.^34^, ^35^

The main limitation of our study is that there are a number of potentially important allergens that are not included on the ISAC chip, such as those from fungi (ISAC has only 6 of the 109 fungal allergens identified in the International Union of Immunological Societies). The use of recombinant allergens means posttranslational modifications, such as proline hydroxylation, which are important for IgE binding in some allergens, such as Ara h 2.^36^ It is possible that the clustering would have been different if additional or alternative allergen components had been available. We also observed that a proportion of children who were not sensitized to any allergen reported symptoms of “allergic” disease, particularly eczema, which was reported in almost 20% of these children. This has been observed in other studies^37^ and reflects the heterogeneity of these diseases in the population.

Sensitization to CG1 (comprising allergens of 8 different protein families of plant origin, all of which, apart from CCD, can be found in pollens) was strongly associated with hay fever (>12-fold compared with those not sensitized to CG1) but not asthma or wheeze. In their classification of pollen allergens, Radauer and Breiteneder^38^ identified that of the 2615 protein families found in seed plants, 29 accounted for more than 150 pollen allergens described. Of the 9 most abundant protein families that were identified as dominating the pollen allergen landscape, all but one clustered in CG1. The remaining common family (EF hand, represented by Bet v 4) clustered to CG3, but only 5 children were sensitized to this allergen. Within individual protein families in CG1, it was often possible to identify a “lead/dominant” allergen to which almost all children were sensitized; for example, among the β-expansins, all children reacted to Phl p 1. Bermuda grass is not found in the United Kingdom, yet 115 subjects had IgE to Cyn d 1. It is likely that this represents cross-reactivity with Phl p 1, which has approximately 67% sequence identity to Cyn d 1. For the profilins, all children were sensitized to Mer a 1 (pollen from annual mercury, a weed commonly seen in Europe) and Hev b 8, and most were also sensitized to Phl p 12 and Bet v 2. For the Bet v 1–like allergens, Bet v 1 was the allergen to which most children were sensitized. It was interesting to observe that for all protein families in CG1, the ISAC array contained at least 1 component known to be present in pollen (www.Allergome.org), and for each individual protein family, the pollen allergen or allergens are frequently the one or ones to which much of the children are sensitized, indicating that these might be the lead allergens. At age 11 years, many children had sIgE to all of Phl p 1, 2, 4, 5, 6, 11, and 12. This is in keeping with the concept of “molecular spreading,” as suggested by the analyses in the German Multicenter Atopy Study 90 cohort.^39^

For CG2, the allergen components were predominantly from dust mites and included the cysteine protease family, where Der p 1 was the allergen to which all children were sensitized, and the group 2 mite allergens, where Der f 2 was the dominant component. Sensitization to CG2 was associated with an approximately 3-fold increase in asthma and a 2-fold increase in hay fever; no association was seen with FEV_1_, but there was a significant increase in airway hyperresponsiveness.

CG3 contained a broad range of protein families, many represented by a single component, and this was the CG to which fewest children were sensitized (8.5%). Sensitization to CG3 was most strongly associated with asthma; FEV_1_ was significantly less in this group (almost 7% less than in those not sensitized to CG3), with a trend for the airways to be more reactive to methacholine. Almost all allergens from domestic pets clustered in this CG. Within the lipocalin protein family, most children were sensitized to Can f 1, and all those sensitized to Can f 2 were sensitized to multiple lipocalins, with a few children sensitized only to Equ c 1 (n = 5) or Fel d 4 (n = 6). Of the 9 children sensitized to Can f 2, all but 1 had asthma. In a recent description of the characteristics of a pediatric population with severe asthma, Konradsen et al^9^ identified that sensitization to the lipocalins Can f 2 and Equ c 1 was associated with severe asthma, whereas for serum albumins (Fel d 2, Can f 3, and Equ c 3), there was no difference between severe and controlled disease. Furthermore, all children sensitized to Can f 2 were in the severe asthma group. It has been suggested that because of sequence similarity with endogenous human lipocalins, these proteins lie at the borderline between self and nonself, which could predispose to T_H_2 immunity.^40^

The patterns we observed can be explained in part by the structural relationships of the allergen components within protein families, as indicated by their sequence similarities (shown in the unrooted dendrograms); that is, within protein families, there are allergens from different biological sources that show significant structural homology. In addition, the collection of protein families within each CG appears to reflect the propensity to develop IgE antibodies to multiple nonhomologous proteins arising from the same biological source. For example, CG1 included almost all of the pollen allergens tested, CG2 included almost all of the dust mite allergens, and CG3 included animal and food allergens. That individual subjects have detectable IgE to multiple members of the same protein family is not in itself a novel observation, and it is not surprising that such allergen components would be in the same CG. As a consequence, each protein family is exclusive to one of the 3 CGs. Indeed, in a study of patients recruited from allergy clinic in Italy, Scala et al^41^ analyzed IgE component results (from an earlier version of ISAC containing 73 allergen components) in more than 3000 patients with IgE reactivity to any member of 3 “panallergen” groups (tropomyosins, profilins, and pathogenesis-related class 10 proteins). They identified a significant direct relationship between different representative molecules within each panallergen group (correlation coefficient usually >0.7). However, when they performed supervised clustering on these data, the components clustered within these panallergen groups, with little evidence of sensitization to more than 1 panallergen. This contrasts with our findings (where pathogenesis-related class 10 protein and profilin components were in the same CG), which might reflect differences in the components included, populations studies, and clustering methodologies used.

The mechanism of development of IgE to a diverse range of proteins from a common biological source is unclear. The nature of T-cell responses to specific environmental allergens might be determined in part by relative expression of different pattern recognition receptors on the surfaces of antigen-presenting cells,^42^ and the role of component-specific IgE on the uptake of the component by dendritic cells is another intriguing finding.^43^ Genetic variation within the HLA locus can also influence the pattern of development of IgE to multiple allergens.^44^, ^45^

In conclusion, our results suggest that distinct patterns of IgE responses to different protein families are associated with different clinical symptoms. Latent variable modeling might help identification of these patterns and development of algorithms that can facilitate better interpretation of multiple sIgE allergen component data. However, our findings need to replicated in other populations before such algorithms can be developed and applied in the clinic.

**Key messages**

• IgE responses to allergen components in a multiplex array (ISAC) cluster into 3 main groups in a population of 11-year-old children.
• These groups contain both homologous proteins from different sources and nonhomologous proteins from the same source.
• IgE responses to clusters of allergens can be differentially associated with respiratory allergies but not with eczema.

## Figures and Tables

**Table I:** Allergen components within each CG

CG	Pfam protein family	ISAC component
CG1	β-Expansins (including N- and C-terminal domains)	C- and N-terminal domains: **nCyn d 1, rPhl p 1**, N-terminal domain only: **rPhl p 2**
	Ribonuclease	**rPhl p 5, rPhl p 6**
	Berberine bridge (FAD binding)	**nPhl p 4**
	Profilin	**rBet v 2**, rHev b 8, **rMer a 1, rPhl p 12**
	Bet v 1 like (PR10)	**rAln g 1, rBet v 1, rCor a 1.0101, rCor a 1.0401**, Mal d 1, rPru p 1, rGly m 4, rAra h 8, rApi g 1
	Ole e 1 family	**rChe a 1, rOle e 1, rPhl p 11**
	CCD	Jug r 2, MUXF3
	Glycosylhydrolase family 28 (polygalacturonase and pectate lyase)	**nPla a 2**, nCry j 1, **nCup a 1**
CG2	MD-2–related lipid recognition (ML) domain (group 2 mite allergens)	rDer p 2, rDer f 2, rLep d 2
	Cysteine protease	nDer p 1, nDer f 1, nAct d 1
	Mite allergen family 5	rBlo t 5
CG3	Prolamin	rAra h 2, nAra h 6, nJug r 3, rPru p 3
	Cupin	rAra h 1, rAra h 3, nGly m 6
	EF hand	**rBet v 4**
	Thaumatin family	nAct d 2
	Lipocalin	rCan f 1, rCan f 2, rEqu c 1, rFel d 4, Mus m1
	Serum albumin	nBos d 6, nCan f 3, nEqu c 3, nFel d 2
	Uteroglobulin-like	rFel d 1
	Kazal-type inhibitor	nGal d 1
	Tropomyosin	nPen m 1, rAni s 3, nBla g 7, rDer p 10
	ATP:guanido phosphotransferase (arginine kinase)	nPen m 2
	Superoxide dismutase	rAsp f 6
	Unique to fungi	Alt a 1
Non-CG	EF-hand	**rPhl p 7**
	Bet v 1 like	rAct d 8
	Cysteine-rich secretory protein family (venom antigen)	rPol d 5, rVes v 5
	Trypsin-like serine protease (peptidase S1)	rCan f 5
	Enolase	rAlt a 6
	Transferrin	rGal d 3
	Glycosyl hydrolases family 17 (β-glucanase)	**rOle e 9**
	γ-Thionin (thionin with O-glycans; Art v 1 family)	nArt v 1
	Cupin	nJug r 1

Pollen allergens are shown in boldface. Allergen nomenclature is based on that of the International Union of Immunological Societies Allergen subcommittee.^33^*PR10*, Pathogenesis-related class 10 proteins.

**Table II:** Association between sensitization to CGs and clinical outcomes

Sensitization to:	Current wheeze (OR [95% CI]), *P* value	Current asthma (OR [95% CI]), *P* value	Current hay fever (OR [95% CI]), *P* value	Eczema (OR [95% CI]), *P* value
CG1	1.48 (0.77-2.84), .24	1.61 (0.84-3.10), .15	**12.79 (6.84-23.90), <.001**	1.10 (0.57-2.13), .78
CG2	**4.19 (2.41-7.30), <.001**	**3.60 (2.05-6.29), <.001**	**2.52 (1.38-4.61), .003**	1.62 (0.91-2.90), .10
CG3	**5.44 (2.42-12.24), <.001**	**8.20 (3.49-19.24), <.001**	1.15 (0.44-3.00), .77	2.00 (0.89-4.49), .09

Values in boldface indicate statistical significance.

## Supplementary data

Online Repository Data

Fig E1

Fig E2

Fig E3

Fig E4

Fig E5

Fig E6

Fig E7

Fig E8

Fig E9

Fig E10

Fig E11
