# Supplementary material for: Patterns of IgE responses to multiple allergen components and clinical symptoms at age 11 years
Source: J Allergy Clin Immunol. 2015 Nov;136(5):1224–31. doi: 10.1016/j.jaci.2015.03.027 (PMC4649774; doi:10.1016/j.jaci.2015.03.027)
Supplement: Online Repository Data [file mmc1.docx]

Patterns of IgE responses to multiple allergen components and clinical symptoms: a birth cohort study

**Online repository**

Angela Simpson MD PhD^1, §^, Nevena Lazic PhD^2,3, §^, Danielle C. M. Belgrave PhD^1,4^, Phil Johnson PhD^1,5^ , Christopher Bishop PhD^2^, E.N. Clare Mills PhD^1,5,^*, Adnan Custovic MD PhD^1,^*

^§^Equal contribution, Joint first authors

*Equal contribution, Joint senior authors

^1^Centre for Respiratory Medicine and Allergy, Institute of Inflammation and Repair, University of Manchester & University Hospital of South Manchester, Manchester, UK

^2^Microsoft Research Cambridge, Cambridge, UK

^3^ currently at Google Inc, USA

^3^Centre for Health Informatics, Institute of Population Health, University of Manchester, Manchester, UK

^5^Manchester Institute of Biotechnology, University of Manchester, Manchester, UK

*Correspondence and requests for reprints:*

Professor Angela Simpson, University of Manchester, ERC Building, Second floor, University Hospital of South Manchester, Manchester M23 9LT, UK

Phone: +44 161 291 5871, Fax: +44 161 291 5730

Email: [*angela.simpson@manchester.ac.uk*](mailto:angela.simpson@manchester.ac.uk)

**Model and Inference**

We analyzed ISAC data for the 71 allergen components with at least three positive tests, and for the 221 children who had a positive test to at least one component. We used two categorical data representations: binary (positive threshold ≥ 0:3) and quaternary (interval boundaries at 0.3, 1, 15).

We investigated a group sensitization model, in which components are assumed to vary in independent non-overlapping groups, and the sensitization to each component in a group j is governed by the latent binary sensitization variable CG_j_ . An allergen component within group j is conditionally independent of components within the same group given CG_j_, and marginally independent of components in other groups. This model is illustrated in Figure E1 Posterior distributions over all latent variables and parameters were computed using Expectation Propagation[E[1](#_ENREF_1)], an algorithm for approximate Bayesian inference. All implementation was done in in Infer.NET[E[2](#_ENREF_2)] .

To determine the number of independent groups and their members, we represented the group of each allergen component d by a multinomial random variable Z_d_, and we inferred all Z_d_ jointly with other latent variables and parameters. We obtained three distinct groups out of a maximum of ten allowed at initialization. Ten allergen components were not strongly assigned as members of any single group, which was not surprising as there were very positive test results for these components. Groups inferred from binary and quaternary data were nearly identical, except for three components (Table E3 and E4).

We assessed the robustness and reproducibility of the solution by training the model on twenty random data subsets of 200 patients. We computed the similarity of each pair of allergen components as the average number of times they were assigned to the same group. The resulting similarity matrices for binary and quaternary data are shown in Figures E2 and E3. Group membership was very stable for binary data, while for quaternary data there was some confusion

between two of the groups.

References

E1. Minka TP. Expectation propagation for approximate Bayesian inference; 2001. Morgan Kaufmann Publishers Inc. pp. 362-369.

E2. Minka T, Winn J, Guiver J, Knowles D Infer .NET 2.4, 2010. Microsoft Research Cambridge.

**Table E1.** Allergen Components to which fewer than three children reacted

| **No children positive** | **1 child positive** | **2 children positive** |
| --- | --- | --- |
| 'Gal d 5' | 'Bos d lactoferrin' | 'Gal d 2' |
| 'Ana o 2' | 'Gad c 1' | 'Bos d 4' |
| 'Fag e 2' | 'Pen m 4' | 'Bos d 5' |
| 'Tri a 14' | 'Ber e 1' | 'Bos d 8' |
| 'Act d 5' | 'Cor a 9' | 'Gly m 5' |
| 'Amb a 1' | 'Ses i 1' | 'Tri a 19.0101' |
| 'Sal k 1' | 'Tri a aA_TI' | 'Pla a 1' |
| 'Asp f 1' | 'Par j 2' | 'Pla l 1' |
| 'Cla h 8' | 'Asp f 3' | 'Hev b 3' |
| 'Bla g 2' | 'Bla g 1' | 'Hev b 6.01' |
| 'Bla g 5' | 'Ani s 1' | 'Ara h 9' |
| 'Api m 1' | 'Hev b 1' | 'Art v 3' |
| 'Api m 4' | 'Cor a 8' | 'Pla a 3' |
| 'Hev b 5' | 'Ole e 7' |  |

**Table E2.** Characteristics of children with and without IgE data at age 11 years

|  | **IgE Data (n=461)** | **No IgE Data (n=352)** |  |
| --- | --- | --- | --- |
|  | **n (%)** | **n (%)** | **p-value** |
| Gender (Male) | 255 (55.3) | 181 (51.4) | 0.121 |
| Age at follow up | 11.49 (0.02) | 11.45 (0.03) | 0.289 |
| Older siblings | 254 (55.1) | 175 (49.7) | 0.074 |
| Maternal smoking (pregnancy) | 53 (11.5) | 34 (9.7) | 0.384 |
| Maternal asthma | 55 (11.9) | 59 (16.8) | 0.071 |
| Paternal asthma | 41 (8.9) | 23 (6.5) | 0.17 |
| Maternal atopy | 255 (55.3) | 213 (60.5) | 0.247 |
| Paternal atopy | 276 (59.9) | 231 (65.6) | 0.196 |
| Current asthma | 58 (12.5) | 46 (13.1) | 0.935 |
| % Predicted FEV | 98.88 (0.53) | 97.70 (0.66) | 0.16 |

**Table E3.** Allergen component groups inferred from binary data.

Highlighted in red are three components for which CG assignment differed between binary and quaternary data representation

| **Variable group** | **Allergen components** |
| --- | --- |
| **CG_1_** | Jug r 2, Cyn d 1, Phl p 1, Phl p 2, Phl p 4, Phl p 5, Phl p 6, Phl p 11, Aln g 1, Cry j 1, Cup a 1, Ole e 1, Pla a 2, Che a 1, Bet v 1, Cor a 1.0101, Cor a 1.0401, Mal d 1, Pru p 1, Gly m 4, Ara h 8, Api g 1, Bet v 2, Hev b 8, Mer a 1, Phl p 12, MUXF3 |
| **CG_2_** | **Act d 1**, Blo t 5, Der f 1, Der f 2, Der p 1, Der p 2, Lep d 2 |
| **CG_3_** | Gal d 1, Pen m 2, Ara h 1, Ara h 2, Ara h 3, Ara h 6, Gly m 6, Bet v 4, Fel d 1, Fel d 4, Can f 1, Can f 2, Equ c 1, Mus m 1, Alt a 1, Asp f 6, Ani s 3, Bla g 7, Der p 10, Pen m 1, Bos d 6, Can f 3, Equ c 3, Fel d 2, Jug r 3, Pru p 3, Act d 2 |
| **Non-CG components** | **Gal d 3**, Jug r 1, Phl p 7, Ole e 9, Art v 1, **Can f 5**, Alt a 6, Pol d 5, Ves v 5, Act d 8 |

**Table E4.** Allergen component groups inferred from quaternary data

Highlighted in red are three components for which CG assignment differed between binary and quaternary data representation

| **Variable group** | **Allergen components** |
| --- | --- |
| **CG_1_** | Jug r 2, Cyn d 1, Phl p 1, Phl p 2, Phl p 4, Phl p 5, Phl p 6, Phl p 11, Aln g 1, Cry j 1, Cup a 1, Ole e 1, Pla a 2, Che a 1, Bet v 1, Cor a 1.0101, Cor a 1.0401, Mal d 1, Pru p 1, Gly m 4, Ara h 8, Api g 1, Bet v 2, Hev b 8, Mer a 1, Phl p 12, MUXF3 |
| **CG_2_** | **Gal d 3**, Blo t 5, Der f 1, Der f 2, Der p 1, Der p 2, Lep d 2 |
| **CG_3_** | Gal d 1, Pen m 2, Ara h 1, Ara h 2, Ara h 3, Ara h 6, Gly m 6, Bet v 4, Fel d 1, Fel d 4, Can f 1, Can f 2, **Can f 5**, Equ c 1, Mus m 1, Alt a 1, Asp f 6, Ani s 3, Bla g 7, Der p 10, Pen m 1, Bos d 6, Can f 3, Equ c 3, Fel d 2, Jug r 3, Pru p 3, Act d 2 |
| **Non-CG components** | Jug r 1, **Act d 1**, Phl p 7, Ole e 9, Art v 1, Alt a 6, Pol d 5, Ves v 5, Act d 8 |

**Table E5**. Assignment of allergens to component groups and allergen isoform sequence accession numbers used in analysis and for generation of dendrograms (Figure E4-E10). *Isoform of allergens is .0101 unless otherwise indicated.

| Component Group | Pfam Protein family | ISAC component (UniProt accession)* | AllFam No | Other protein family members from Allfam used in dendrograms (Fig E3-E9) |
| --- | --- | --- | --- | --- |
| CG1 | β-Expansins (including N- and C-terminal domain) | C- and N-terminal: nCyn d 1 (O04701),  rPhl p 1 (Q40967),  N-terminal only: rPhl p 2 (P43214) | 34 (22 N-terminal, 12 C-terminal domain) | C- and N-terminal: Dac g 1, Hol l1, Lol p 1, Ory s 1, Pas n 1, Pha a 1, Phl p 1, Poa p 1, Tri a 1, Zea m 1  N-terminal only : Cyn d 15, Cyn d 2, Dac g 2, Dac g 3, Lol p 2, Lol p 3, Phl p 3, Poa p 2, Tri a 2, Zea m 3  C-terminal only : Act d 5, |
|  | Profilin | rBet v 2 (P25816), rHev b 8 (Q9STB6), rMer a 1 (O49894), rPhl p 12 (O24650) | 48 | Ama r 2, Amb a 8, Ana c 1, Api g 4, Ara h 5, Art v 2, Bra n 8, Cap a 2, Che a 2, Cit la 2, Cit s 2, Cor a 2, Cro s 2, Cuc m 2, Cyn d 12, Dau c 4, Fra a 4, Gly m 3, Hel a 2, Hom s profilin, Hor v 12, Hum j 2, Lit c 1, Lyc e 1, Mal d 4, Man i 3, Mus a 1, Nic t 8, Ole e 2, Par j 3, Pet c 2, Pho d 2, Pru av 2, Pru du 4, Pru p 4, Pyr c 4, Ric c 8, Sal k 4, Sola t 8, Tri a 12, Vit v 4, Zea m 12. |
|  | Bet v 1 | rAln g 1 (P38948), rBet v 1 ( P15494), rCor a 1 (Q08407), rCor a 1.0401 (Q9SWR4), Mal d 1.0108 (Q9SYW3), rPru p 1 (Q2I6V8), rGly m 4 (P26987), rAra h 8 (Q6VT83), rApi g 1 (P49372) | 30 | Act c 8, Act d 11, Act d 8, Bet ch 1, Bet co 1, Bet le 1, Bet n 1, Bet p 1, Car b 1, Cas s 1, Cor he 1, Cau c 1, Fag s 1, Fra a 1, Lyc e 4, Ost c 1, Pru ar 1, Pru av 1, Pyr c 1, Que e 1, Rub i 1, Vig r 1 |
|  | Glycosylhydrolase family 28 (Poly-galacturonas e and Pectate lyase) | nPla a 2 (Q6H9K0) nCry j 1 (P18632), nCup a 1 (A0T2M2, Q9SCG9) | 10, 11 | Asp n pectinase, Bra n PG, Cha o 2, Cry j 2, Jun a 2, Lil l PG, Lyc e PG, Phl p 13, Pla or 2, Zea m 13  Amb a 1, Art v 6, Asp f PL, Cha o 1, Cup s 1, Jun a 1, Jun v 1, Pen c 32 |
|  | Ole e 1 family | rChe a 1 (Q8LGR0), rOle e 1 (P19963), rPhl p 11 (Q8H6L7) | 10 | Cro s 1, Fra e 1, Lig v 11, Lol p 11, Pla l 1, Sal k 5, Zea m 13. |
|  | Ribonuclease | rPhl p 5 (Q9SBE0), rPhl p 6 (P43215) | 10 | Dac g 1, Hol l 5, Hor v 5, Lol p 5, Pha a 5, Poa p 5, Sec c 5, Tri a 5 |
|  | Berberine bridge | nPhl p 4 (B2ZWF1) | 7 | Asp f IAO, Cyn d 4, Hor v 4, Lol p 4, Sec c 4, Tri a 4 |
|  | CCD | Jug r 2, MUXF3 | Not applicable | Not relevant |
| CG2 | Cysteine protease | nAct d 1 (A5HIJ1), nDer f 1 (P16311), nDer p 1.0111 (P08176) | 11 | Ana c 2, Blo t 1, Car p 1, Car p chymopapain, Carp p endoproteinase, Eur m 1, Gly m Bd 30k, Pso o 1 |
|  | MD-2-related lipid-recognition (ML) domain (Group 2 mite allergens) | rDer f 2 (Q8WQK5), rDer p 2.0104 (P49278), rLep d 2 (P80384) | 10 | Blo t 2, Der f 22, Der s 2, Eur m 2, Gly d 2, Pso o 2, Tyr p 2 |
|  | Mite allergen family 5 | rBlo t 5 (O96870) | 6 | Blo t 21, Der f 5, Der p 21, Der p 5, Lep d 5 |
| CG3 | Prolamin | rAra h 2.01 (A5Z1Q8), nAra h 6 (Q647G9), nJug r 3 (C5H617), rPru p 3.01 (Q5RZZ3), | 82 | Amb a 6, Ana o 3, Api g 2, Ara h 7,Ara h 9, Ara t 3, Art v 3, Ber e 1, Bra j 1, Bra n 1, Bra o 3, Bra r 1, Bra r 3, Car i 1, Cit s 3, Cor a 14, Cor a 8, Dau c 3, Fag e 10kD, Fag e 2, Fag t 2, Fra a 3, Gly m 2S Albumin, Hel a 2S Albumin, Hel a 3, Hev b 12, Hor v 14, Hor v 15, Hor v 20, Hor v BDAI, Hor v BTI, Jug n 1, Jug r 1, Lac s 1, Len c 3, Lup a delta_Conglutin, Lup an delta_Conglutin, Lyc e 3, Mal d 3, Mor n 3, Ory s 14, Ory s 17kD, Ory s 19kD, Ory s aA_TI, Par j 1, Par j 2, Pha v 3, Pis v 1, Pla a 3, Pla or 3, Pru ar 3, Pru av 3, Pru d 3, Pru du 3, Pyr c 3, Ric c 1, Rub i 3, Ses i 1, Ses i 2, Sin a 1, Sin a 3, Tri a 14, Tri a 15, Tri a 21, Tri a 28, Tri a 29,Tri a 30, Tri a alpha_Gliadin, Tri a CM16, Tri a gamma_Gliadin, Tri a Gliadin, Tri a LMW Glu, Tri td 14, Tri td aA_TI, Vit v 1, Zea m 14 |
|  | EF-Hand | rBet v 4 (nQ39419) | 63 | Aln g 4, Amb a 10, Amb a 9, Ang j 1, Ani s Troponin, Art v 5, Bet v 3, Bla g 6, Bla g 8, Bos d 3, Bos d PRVB, Bra n 4, Bra n 7, Bra r 4, Bra r 7, Che a 3, Clu h 1, Cra c 4, Cra c 6, Cup a 4, Cyn d 7, Cyp c 1, Equ c PRVB, Evy j 1, Gad c 1, Gad m 1, Gal d PRVB, Hom a 6, Hom s 4, Jun o 4, Kat p 1, Lep w 1, Lit v 3, Lit v 4, Mer ca 1, Mer mr 1, Ole e 3, Ole e 8, Onc m 1, Par j 4, Par ol 1, Pen m 3, Pen m 4, Pen m 6, Per a 6, Phl p 7, Pon l 4, Ran e 1, Ran e 2, Sal s 1, Sar m 1, Sar sa 1, Sco j 1, Sco s 1, Seb m 1, Sus s PRVB, Syr v 3, The c 1, Thu a 1 |
|  | Tropomyosin | nPen m 1 (A1KYZ2), rAni s 3 (Q9NAS5), nBla g 7 (Q9NG56), rDer p 10 (O18416) | 47 | Bal r 1, Blo t 10, Cha f 1, Chi k 10, Chi o 1, Cra c 1, Cra g 1, Der f 10, Dro m 7, Eri i 1, Eri s 1, Eup p 1, Eup s 1, Hal d 1, Hal di 1, Hel as 1, Hom a 1, Lep d 10, Lep s 1, Lit v 1, Mar j 1, Met e 1, Myt e 1, Oct v 1, Omm b 1, Ora o 1, Pan b 1, Pan e 1, Pan s 1, Par c 1, Pat y 1, Pen a 1, Per a 7, Per f 7, Per v 1, Por tr 1, Scy s 1, Sep e 1, Sep l 1, Sin c 1, Tod p 1, Tur c 1, Tyr p 10 |
|  | Cupin | rAra h 1 (P43238), rAra h 3 (Q5I6T2), nGly m 6.0101 and 6.0201 (P04776, P04405) | 43 | Ana o 1, Ana o 2, Ara h 3.02, Ara t GLP, Ber e 2, Car i 4, Cor a 11, Cor a 9, Fag e 1, Fag e 19kD, Fag t 1, Gly m 5, Gly m Bd28K, Gly m Bd60K, Gos h Vicilin, Jug n 2, Jug r 2, Jug r 4, Len c 1, Lup a 1, Lup a alpha_Conglutin, Lup an 1, Lup an alpha_Conglutin, Ory s GLP52, Ory s GLP63, Ory s NRA, Pha v Phaseolin, Pis s 1, Pis s 2, Pis v 2, Pis v 3, Pis v 5, Pru du 6, Ses i 3, Ses i 6, Ses i 7, Sin a 2, Vig r 8S Globulin, Zea m G1, Zea m G2 |
|  | Lipocalin | rCan f 1 (O18873), rCan f 2 (O18874), rEqu c 1 (Q95182), rFel d 4 (Q5VFH6), nMus m 1 (P02762) | 25 | Aca s 13, Blo t 13, Bos d 2, Bos d 5, Bub b BLG, Can f 6, Cap h BLG, Cav p 2, Cav p 3, Der f 13, Der p 13, Equ c BLG, Fel d 7, Hom s TL, Lep d 13, Ovi a BLG, Per a 4, Ran t BLG, Rat n 1, Tyr p 13 |
|  | Thaumatin family | nAct d 2 (P83958, P81370) | 15 | Cap a 1, Cry j 3, Cup a 3, Cup s 3, Jun a 3, Jun v 3, Lyc e PR23, Mal d 2, Nic t Osmotin, Ole e 13, Pru av 2, Pru p 2, Tri a TLP  Vit v TLP |
|  | Serum albumin | nBos d 6 (B0JYQ0), nCan f 3 (P49822), nEqu c 3 (P35747), nFel d 2 (P49064) | 13 | Cap h GSA, Equ as DSA, Equ c 3, Fel d 2  Gal d 5, Hom s HSA, Mus m MSA, Ory c RSA  Ovi a SSA, Rat n RSA, Sus s PSA |
|  | ATP:guanido phosphotransferase (Arginine kinase) | nPen m 2 (E7CGC2, C7E3T4) | 11 | Bla g 9, Chi o 2, Cra c 2, Der p 20, Hom g 2, Lit v 2, Met e 2, Pen m 2, Per a 9, Plo i 1, Scy s 2 |
|  | Superoxide dismutase | rAsp f 6 (Q92450) | 8 | Alt a MnSOD, Cand a MnSO, Mala s 11 |
|  | Kazal-type inhibitor | nGal d 1 (B6V1G0, P01005) | 5 | Ana p 1, Ans a 1, Mel g 1, Str c 1 |
|  | Uteroglobulin-like | rFel d 1 (P30438) | 1 | None |
|  | Unique to fungi | Alt a 1 (P79085) | 1 | None |
| Non-CG | EF-hand | rPhl p 7 (O82040) | 63 |  |
|  | Bet v 1 | rAct d 8 (D1YSM5) | 30 |  |
|  | Cysteine-rich secretory protein family (Venom antigen) | rPol d 5 (P81656), rVes v 5 (Q05110) | 27 | Cte f 2, Dol a 5, Dol m 5, Glo m 5, Pac c 3, Pol a 5, Pol e 5, Pol f 5, Pol g 5, Poly s 5, Sim vi 1, Sol i 3, Sol r 3, Tab y 5, Ves f 5, Ves g 5, Ves m 5, Ves p 5, Ves s 5, Ves vi 5, Vesp c 5, Vesp m 5, Vesp ma 5 |
|  | Trypsin-like serine protease (Peptidase S1) | rCan f 5 (P09582) | 18 | Api m 7, Bla g Trypsin, Blo t 3, Blo t 6, Bom p 4, Bom t 4, Bos d Thrombin, Cul n 11, Der f 3, Der f 6, Der p 3, Der p 9, Eur m 3, Hom s PSA, Pol d 4,Tyr p 3 |
|  | Enolase | rAlt a 6 (Q9HDT3) | 12 | Asp f 22, Bea b Enol, Cand a Enolase, Cla h 6, Cur l 2, Pen c 22, Rho m 1 |
|  | Transferrin | rGal d 3 (P02789) | 8 | Ana p 3, Bos d Lactoferrin, Gal d 3, Hom s Lactoferrin, Mel g 3, Rat n Transferrin, Str c 3,  Str pu Vitellogenin |
|  | Glycosyl hydrolases family 17 (β-glucanase) | rOle e 9 (Q94G86) | 7 | Cap a Glucanase, Hev b 2, Lyc e Glucanase, Mus a 5, Ole e 9, Sola t Glucanase, Vit v Glucanase |
|  | γ-Thionin (thionin with O-glycans; Art v 1 family) | nArt v 1 (Q84ZX5) | 2 | Amb a 4 |
|  | Cupin | nJug r 1 (P93198) | 43 | Ana o 1, Ana o 2, Ara h 4, Ara t GLP, Ber e 2, Car i 4, Cor a 11, Cor a 9, Fag e 1, Fag e 19kD, Fag t 1, Gly m 5, Gly m Bd28K, Gly m Bd60K, Gos h Vicilin, Jug n 2, Jug r 2, Jug r 4, Len c 1, Lup a 1, Lup a alpha_Conglutin, Lup an 1, Lup an alpha_Conglutin, Ory s GLP52, Ory s GLP63, Ory s NRA, Pha v Phaseolin, Pis s 1, Pis s 2, Pis v 2, Pis v 3, Pis v 5, Pru du 6, Ses i 3, Ses i 6, Ses i 7, Sin a 2, Vig r 8S Globulin, Zea m G1, Zea m G2 |

**Table E6.** Characteristics of 461 children with IgE data at age 11 assigned to different component groups. We assigned each child as sensitised or not to each of the CGs using posterior cut-off threshold of 0.5, Sensitisations were not mutually exclusive, and of those children sensitised to the CGs, 99 were sensitised to one CG only, 34 were sensitised to 2 CGs and 16 were sensitised to all 3 CGs.

|  | **Non-sensitised Group (n=240)** | **Sensitised non-CG (n=72)** | **Positive CG1**  **(n=78)** | **Positive CG2 (n=98)** | **Positive CG3 (n=39)** |
| --- | --- | --- | --- | --- | --- |
|  | **n (%)** | **n (%)** | **n (%)** | **n (%)** | **n (%)** |
| **Male** | 108/240 (45.0) | 45/72 (62.5) | 52/78 (66.7) | 65/98 (66.3) | 27/39 (69.2) |
| **Asthma** | 20/231 (8.7) | 5/72 (6.9) | 23/76 (30.3) | 35/96 (36.5) | 20/38 (52.6) |
| **Current Wheeze** | 20/240 (8.3) | 8/71 (11.3) | 30/77 (39.0) | 46/96 (47.9) | 24/37 (64.9) |
| **Current Hay fever** | 11/230 (4.8) | 20/72 (27.8%) | 52/76 (68.4) | 44/95 (46.3) | 20/36 (55.6) |
| **Current Eczema** | 46/239 (19.2) | 16/71 (22.5) | 19/76 (25.0) | 26/95 (27.4) | 12/37 (32.4) |
|  |  |  |  |  |  |
|  | **Mean**  **(95% CI)** | **Mean**  **(95% CI)** | **Mean**  **(95% CI)** | **Mean**  **(95% CI)** | **Mean**  **(95% CI)** |
| **FEV_1_**  **% Predicted** | 99.53  (98.22 - 100.83) | 99.07  (96.59 - 101.56) | 97.59  (94.52 - 100.66) | 96.83  (94.18 - 99.49) | 92.82  (88.40 - 97.23) |
| **Normalised DRR methacholine** | 5.20  (4.81 - 5.59) | 4.69  (3.91 - 5.47) | 3.54  (2.84 - 4.25) | 2.80  (2.19 - 3.42) | 2.89  (1.58 - 4.29) |
| **eNO ppb**  **(Geometric Means)** | 8.37  (7.88 - 8.88) | 13.53  (11.16 - 16.40) | 23.17  (18.10 - 29.68) | 31.05  (25.65 - 37.57) | 35.96  (27.53 - 46.97) |

**Table E7.** Patterns of co-morbid diseases amongst children assigned to each sensitisation group

We assigned each child as sensitised or not to each of the CGs using posterior cut-off threshold of 0.5, Sensitisations were not mutually exclusive, and of those children sensitised to the CGs, 99 were sensitised to one CG only, 34 were sensitised to 2 CGs and 16 were sensitised to all 3 CGs.

|  | **Non-sensitised Group (n=220)** | **Sensitised non-CG (n=69)** | **Positive CG1**  **(n=76)** | **Positive CG2 (n=94)** | **Positive CG3 (n=35)** |
| --- | --- | --- | --- | --- | --- |
|  | **n (%)** | **n (%)** | **n (%)** | **n (%)** | **n (%)** |
| **Hay fever only** | 6 (2.7) | 9 (13) | 22 (28.9) | 15 (16.0) | 3(8.6)) |
| **Eczema only** | 47 (21.4) | 16 (23.2) | 2(2.6) | 13 (13.8) | 1(2.9) |
| **Asthma only** | 8 (3.6) | 3 (4.3) | 8 (10.5) | 12 (12.8) | 6 (17.1) |
| **Hay fever and asthma** | 1 (0.5) | 0 (0) | 7 (9.2) | 10 (10.6) | 3 (8.6) |
| **Asthma and eczema** | 7 (3.2) | 3 (4.3) | 6 (7.9) | 13 (13.8) | 6 (17.1) |
| **Eczema and hay fever** | 3 (1.4) | 6 (8.7) | 13 (17.1) | 9 (9.6) | 4(11.4) |
| **Asthma, eczema, hay fever** | 1 (0.5) | 2 (2.9) | 10 (13.2) | 14 (14.9) | 10(28.6) |
| **Disease free** | 147 (66.8) | 30(43.5) | 8 (10.5) | 15 (16.0) | 2(5.7) |

Figure legends

**Figure E1**: Group sensitization model, where sensitization to each component is governed by one of the latent binary sensitization variables CG_j_ .

**Figure E2:** Pairwise component similarity matrix, with allergen components sorted according to the inferred groups using binary data. The similarity of a pair of components was computed as the average number of times they were assigned to the same group across twenty inference runs on random subsets of 200 patients. Grey levels are scaled such that black corresponds to similarity 0 and white to maximum similarity 1.

**Figure E3:** Pairwise component similarity matrix, with allergen components sorted according to the inferred groups, using quaternary data, as per Figure E2.

**Figure E4.** (A) Dendrogram showing sequence relations within the Profilin AllFam protein family with IgE-reactive sequences of ISAC components coloured. The allergens present in this family are described further in Tables 1 and E5. (B). Venn diagram showing the co-occurrence of CG1 patient serum IgE-reactivity within the Profilin protein family The number of patient sera with significant reactivity to each ISAC component is shown. Allergen isoforms are .0101 unless otherwise indicated.

**Figure E5**. (A) Dendrogram showing sequence relations within the Expansin AllFam protein family with IgE-reactive sequences of ISAC components coloured. The allergens present in this family are described further in Tables 1 and E5.(B) Venn diagram showing the co-occurrence of CG1 patient serum IgE-reactivity within the Expansin protein family. The number of patient sera with significant reactivity to each ISAC component is shown. Allergen isoforms are .0101 unless otherwise indicated.

**Figure E6.** (A) Dendrogram showing sequence relations within the Bet v 1 AllFam protein family with IgE-reactive sequences of ISAC components coloured. The allergens present in this family are described further in Tables 1 and E5.(B) Venn diagram showing the co-occurrence of CG1 patient serum IgE- reactivity within the Bet v 1 protein family. The number of patient sera with significant reactivity to each ISAC component is shown. Allergen isoforms are .0101 unless otherwise indicated.

**Figure E7**. (A) Dendrogram showing sequence relations within the Pectate lyase AllFam protein family with IgE-reactive sequences of ISAC components coloured. The allergens present in this family are described further Tables 1 and E5.(B) Venn diagram showing the co-occurrence of CG1 patient serum IgE reactivity within the Pectate lyase protein family. The number of sera with significant reactivity to each ISAC component is shown. Allergen isoforms are .0101 unless otherwise indicated.

**Figure E8**. (A) Dendrogram showing sequence relations within the Ole e 1-like AllFam protein family with IgE-reactive sequences of ISAC components coloured. The allergens present in this family are described further in Tables 1 and E5.(B) Venn diagram showing the co-occurrence of CG1 patient serum IgE reactivity within the Ole e 1-like protein family. The number of patient sera with significant reactivity to each ISAC component is shown. Allergen isoforms are .0101 unless otherwise indicated

**Figure E9**. Venn diagrams showing the co-occurrence of CG2 patient serum IgE-reactivity with protein families identified from ISAC data (Tables 1 and E5). The number of patient sera with significant reactivity to each ISAC component is shown. Allergen isoforms are .0101 unless otherwise indicated.

**Figure E10**. Venn diagrams showing the co-occurrence of CG3 patient serum IgE-reactivity with protein families identified from ISAC data (Tables 1 and E5). The number of patient sera with significant reactivity to each ISAC component is shown. Allergen isoforms are .0101 unless otherwise indicated.

**Figure E11.** Venn Diagram illustrating assignment of study population. The number of children sensitised to each combination of component groups CG1, CG2, CG3 is shown
