# Supplementary figures and images for: Patterns of IgE responses to multiple allergen components and clinical symptoms at age 11 years
Source: J Allergy Clin Immunol. 2015 Nov;136(5):1224–31. doi: 10.1016/j.jaci.2015.03.027 (PMC4649774; doi:10.1016/j.jaci.2015.03.027)

**Figure E1**:


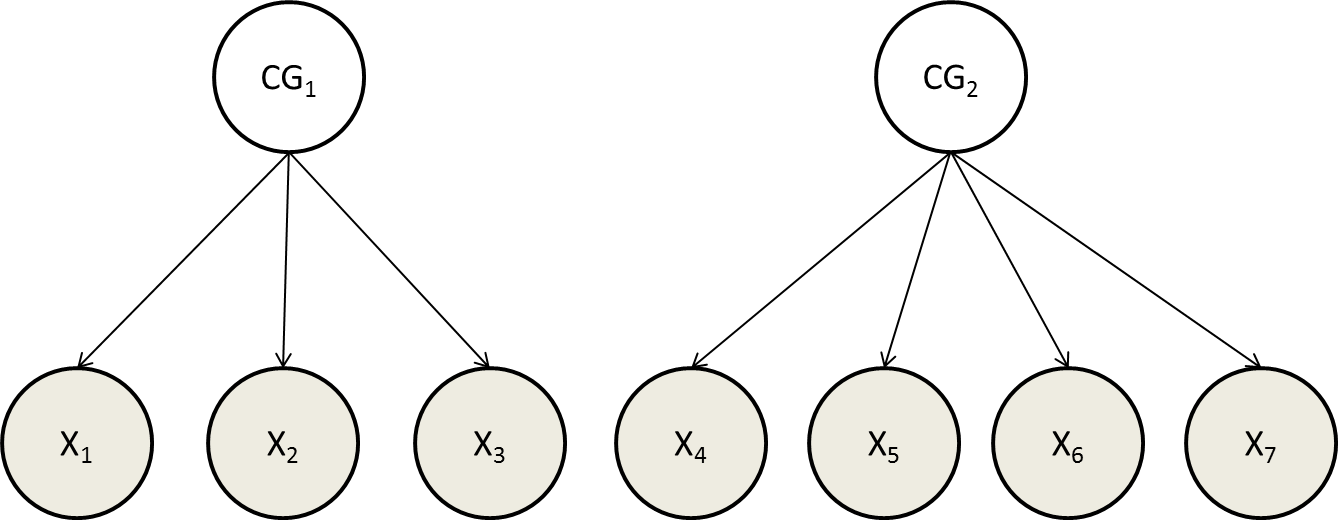

Supplement: Fig E1 [file mmc2.docx]

**Figure E2**


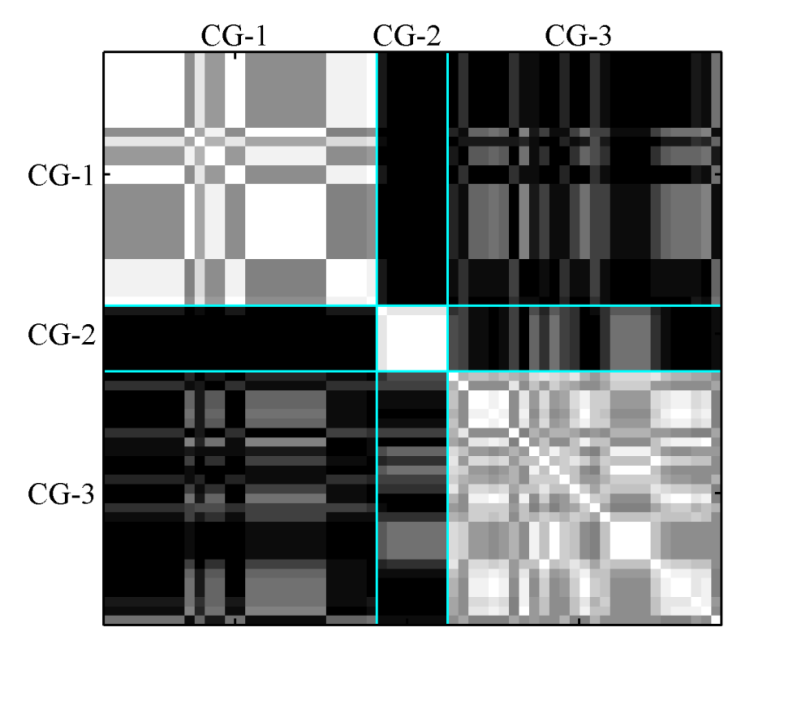

Supplement: Fig E2 [file mmc3.docx]

**Figure E3**


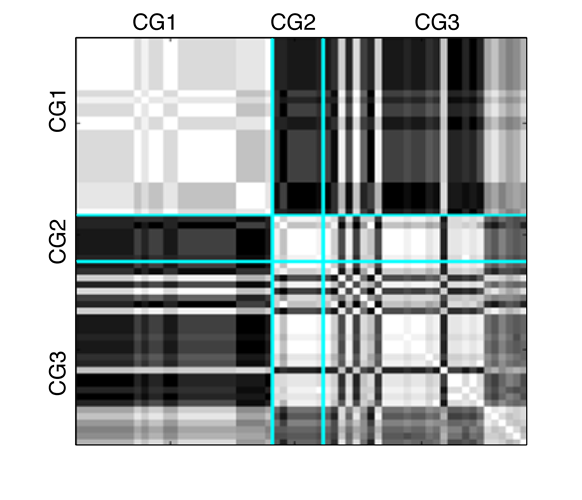

Supplement: Fig E3 [file mmc4.docx]

**Figure E11**


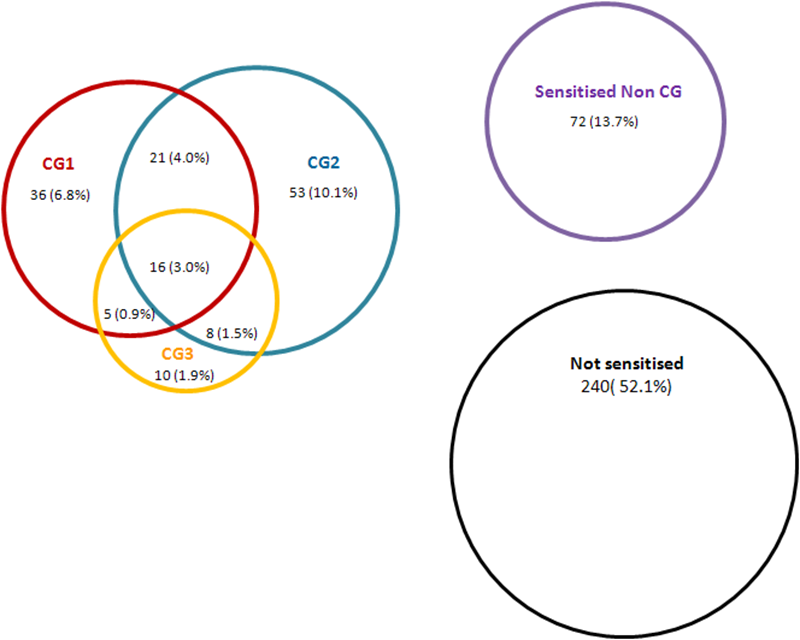

Supplement: Fig E11 [file mmc12.docx]
