# Supplementary material for: Patterns of IgE responses to multiple allergen components and clinical symptoms at age 11 years
Source: J Allergy Clin Immunol. 2015 Nov;136(5):1224–31. doi: 10.1016/j.jaci.2015.03.027 (PMC4649774; doi:10.1016/j.jaci.2015.03.027)
Supplement: Fig E4 [file mmc5.docx]

**Figure E4**
